# Functioning transferred free muscle innervated by part of the vascularized ulnar nerve connecting the contralateral cervical seventh root to themedian nerve: Case report

**DOI:** 10.1186/1749-7221-2-18

**Published:** 2007-09-21

**Authors:** Ryosuke Kakinoki, Ryosuke Ikeguchi, Ken Nakayama, Takashi Nakamura

**Affiliations:** 1 Department of Orthopedic Surgery & Rehabilitation Medicine, Graduate School of Medicine, Kyoto University, Kyoto, Japan

## Abstract

**Background:**

The limited nerve sources available for the reconstruction and restoration of upper extremity function is the biggest obstacle in the treatment of brachial plexus injury (BPI). We used part of a transplanted vascularized ulnar nerve as a motor source of a free muscle graft.

**Case presentation:**

A 21-year-old man with a left total brachial plexus injury had received surgical intercostal nerve transfer to the musculocutaneous nerve and a spinal accessory nerve transfer to the suprascapular nerve in another hospital previously. He received transplantation of a free vascularized gracilis muscle, innervated by a part of the transplanted vascularized ulnar nerve connecting the contralateral healthy cervical seventh nerve root (CC7) to the median nerve, and recovered wrist motion and sensation in the palm. At the final examination, the affected wrist could be flexed dorsally by the transplanted muscle, and touch sensation had recovered up to the base of each finger. When his left index and middle fingers were touched or scrubbed, he felt just a mild tingling pain in his right middle fingertip.

**Conclusion:**

Part of the transplanted vascularized ulnar nerve connected to the contralateral healthy cervical seventh nerve root can be used successfully as a motor source and may be available in the treatment of patients with BPI with scanty motor sources.

## Background

The limited nerve sources available for the reconstruction and restoration of upper extremity function is the biggest obstacle in the treatment of brachial plexus injury (BPI). Recently, there have been several techniques invented to solve this. One is the use of the contralateral healthy cervical seventh nerve root (CC7) reported by Gu et al. in 1992 [1]. They used this as a donor nerve to reconstruct the median nerve function of the affected arm. Another is the use of a part of the ulnar nerve transfer for reinnervation of the biceps brachii muscle for the treatment of upper type BPI reported by Oberlin et al. in 1994 [2]. Several authors have reported excellent outcomes for nerve recovery with the use of these techniques without leaving functional loss of the donor nerves [3-7].

We describe a 21-year-old patient with BPI who received a vascularized free gracilis muscle transfer [8] innervated by a part of the vascularized ulnar nerve connecting CC7 to the affected median nerve. This successfully restored wrist motion and sensation to the palm.

## Case presentation

**Figure 1:**
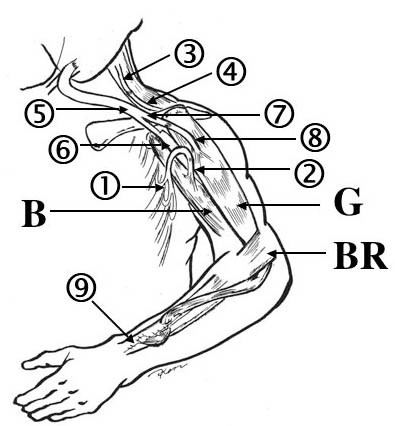
**Scheme of the operative procedures of this case**. Left third to fifth intercostal nerves () were transferred to the left musculocutaneous nerve () and the left accessory nerve () was connected to the ipsilateral suprascapular nerve (). These procedures had been done previously in another hospital. The left ulnar nerve (), vascularized by the superior ulnar collateral vessels and ulnar vessels, was transplanted between the right seventh cervical nerve root (CC7) and left median nerve () (the first stage of the surgery). A vascularized gracilis muscle (G) was harvested from the right thigh and transplanted to the left arm. The muscle, innervated by part of the vascularized ulnar nerve () and nourished by the transverse cervical vessels, was connected to the clavicle and the extensor carpi radialis brevis tendon () (the second stage of the surgery). , anterior branch of the obturator nerve; B. biceps brachii muscle; BR, brachioradialis muscle.

A 21-year-old man sustained contusion injuries to both lungs and the brain in a motorcycle accident. He was transferred to a hospital, where he was treated for the lung and brain injuries. As his consciousness recovered, his left arm was found to be completely paralyzed. He was diagnosed as having a total left BPI, and underwent surgery for this three months after the injury. During surgery, the left third, fourth and fifth intercostal nerves were transferred to the left musculocutaneous nerve [9], the sixth and seventh intercostal nerves to the left thoracodorsal nerve, and the left accessory nerve to the left suprascapular nerve [10] (Fig. 1).

Twelve months after the BPI surgery performed in the previous hospital, he visited our clinic complaining of complete loss of sensation and motor function distal to the left elbow joint. Reasonable recovery of the shoulder and elbow joints was seen from a physical examination. The left elbow flexion range was 100° against gravity and the abduction and flexion ranges of the left shoulder against gravity were 80° and 85°, respectively. However, the elbow extension was M0 according to the Medical Research Council Scale (MRCS) and the range of left shoulder extension was 15°, which was mostly achieved by motion of the left scapula. No active motion was observed in the left wrist and fingers. The patient demonstrated complete numbness in the area innervated by the left C5-T1 nerve roots. He had slight stiffness in the MP joints of the left index finger to the little finger, and started rehabilitation exercises to soften the joints. No other joints of his left upper extremity showed restriction of the range of motion in the passive movement. Elbow flexion was recovered to M4 level according to MRCS at 15 months after surgery thanks to muscle-strengthening exercises.

**Figure 2:**
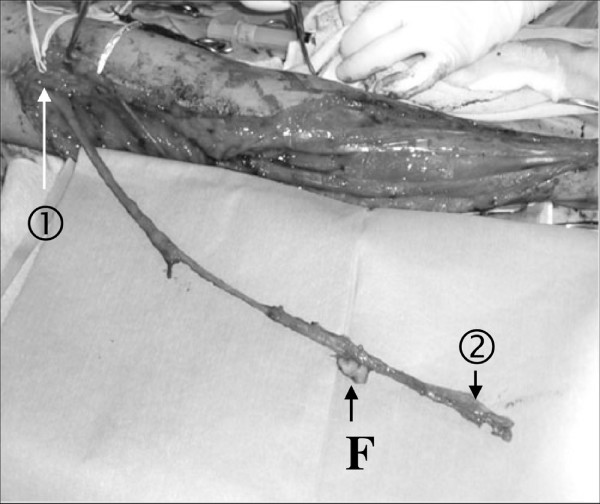
**The harvested vascularized ulnar nerve**. The ulnar nerve, vascularized by the superior ulnar collateral vessels proximally (arrow), was harvested containing the ulnar vessels (arrow heads) distally. A monitor flap (F) was taken attached to the ulnar vessels.

**Figure 3:**
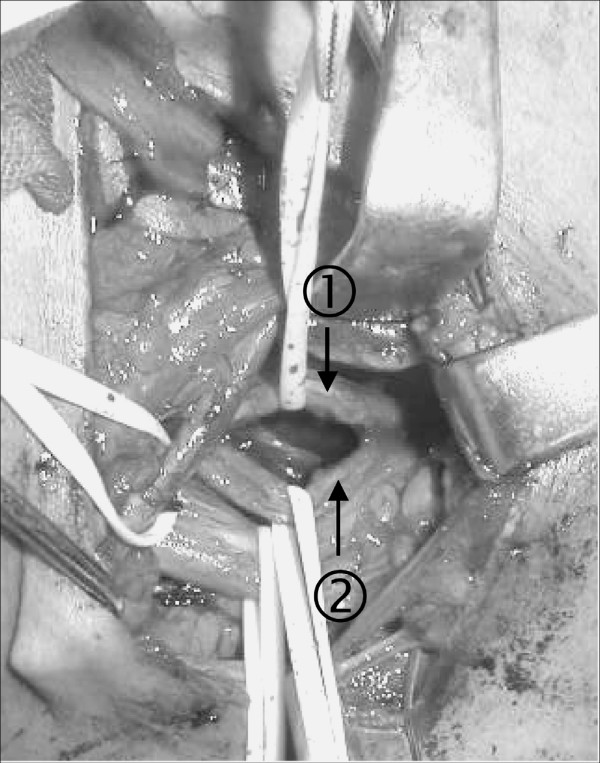
**Preparation of the donor C7 root**. The contralateral healthy seventh cervical nerve root was divided into the posterosuperior () and anteroinferior () portions. The posterosuperior portion was sectioned and connected to the vascularized ulnar nerve.

Eighteen months after the injury, he underwent reconstructive surgery to his left wrist and hand. As the first step of the reconstruction, the CC7 was transferred to the median nerve through a vascularized ulnar nerve taken from the affected arm. The left ulnar nerve was elevated based on the superior ulnar collateral vessels, including the ulnar vessels distally. A monitor skin flap (2.5 × 1 cm) supplied by perforators arising from the ulnar vessels was also harvested from the distal third of the forearm together with the ulnar nerve. The ulnar nerve and vessels were sectioned at the wrist level distally. The ulnar nerve was also sectioned just proximal to the insertion of the superior ulnar collateral vessels into the ulnar nerve in the upper arm level proximally (Fig. 2). The median nerve was sectioned at the same level of the proximal section of the ulnar nerve. The proximal stump of the transected ulnar nerve segment was approximated to the distal stump of the median nerve. The distal stump of the ulnar nerve segment including the ulnar vessels was transferred to the right neck through a subcutaneous tunnel and approximated the posterosuperior portion of the right seventh cervical nerve root as described by Songcharoen et al. [5] (Fig. 3). The distal stump of the ulnar artery accompanying the transplanted ulnar nerve segment was approximated to the transverse cervical artery in the right neck followed by ligation of the distal stumps of the ulnar veins. After surgery, the monitor flap survived completely. The patient showed transient paresthesia in the tips of right index and middle fingers, which lasted for two months. Grip strength was not affected. The sensory recovery of the affected limb was rapid, and touch sensation was restored to the midpalm level of the affected hand by four months after the CC7 transfer.

**Figure 4:**
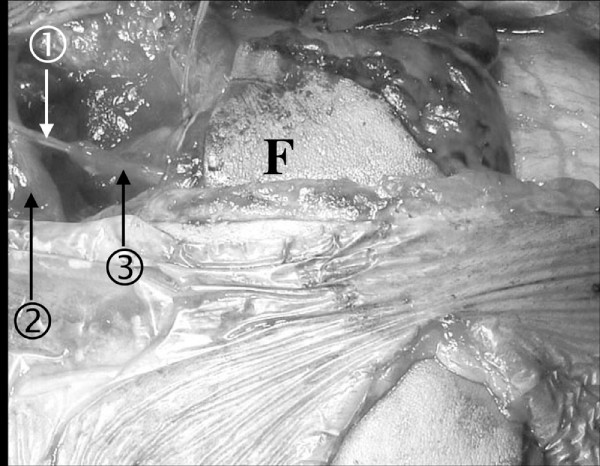
**Par of the vascularized ulnar nerve transfer**. Two funiculi () were harvested from the vascularized ulnar nerve () and connected to the obturator nerve () innervating the transplanted gracilis muscle (F).

As the second stage of the reconstruction, the free vascularized gracilis muscle taken from the right medial thigh was transplanted to the left upper arm four months after the CC7 transfer. The proximal stump of the gracilis was joined to the clavicle with bone anchors and to the trapezius with 1-0 Ethicon sutures. The distal stump of the transplanted muscle was passed under the brachioradialis muscle, stretched maximally and sutured to the extensor carpi radialis brevis tendon in interlacing fashion, maintaining the shoulder joint at 60° abduction and 30° flexion, and the elbow joint at 45° flexion. The tension of the transplanted muscle was set higher than normal, because a slight flexion contracture of the elbow joint would help the patient bend the elbow joint. The medial circumflex femoral vessels of the transplanted gracilis muscle were approximated to the left transverse cervical vessels, as the thoracoacromial vessels were not suitable to microvascular anastomosis because of severe scarring. In the left anterior axillary area, two funiculi of the ulnar nerve (about 10% thickness of the original nerve) were separated from the vascularized ulnar nerve–having been transplanted in the previous operation–and taken proximally for about 1.5 cm. The proximally based partial ulnar nerve stump was approximated to the anterior branch of the obturator nerve innervating the free gracilis muscle (Fig. 4). The stump size of the two funiculi of the ulnar nerve matched that of the branch of the obturator nerve. The distance between the site of the neurorraphy and the entrance of the obturator nerve to the gracilis muscle belly was about 3 cm.

**Figure 5:**
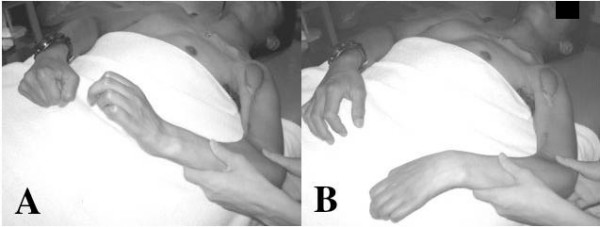
**A and B -Dorsiflexion of the wrist after 15 months after the free gracilis muscle transfer**. Fifteen months after the muscle graft, the dorsiflexion of the left wrist was 35° against gravity with the elbow joint held at a 30° -flexion position when the patient tensed the flexor muscles of his right wrist and fingers. A: Dorsiflexion of the left wrist with synchronous flexion of the right wrist and fingers. B: Palmar flexion of the wrist by the gravity.

Postoperative recovery was uneventful. After the muscle graft operation, the patient's sensation in the left palm was not compromised and showed continuous further recovery, even though almost 10% of the nerve fibers had been taken to innervate the transplanted muscle. Contraction of the transplanted muscle was observed eight months after the transplantation. Fifteen months after the muscle graft, the dorsiflexion of the left wrist was 35° against gravity with the elbow joint held fully extended (approximately at a 30° -flexion position) when the patient applied forces to the flexor muscles of his right wrist and ulnar fingers (mainly, right and little fingers). His right fist formation and slight palmar wrist flexion was synchronously sccompanied by the dorsiflexion of his left wrist (Fig. 5A and 5B). The left elbow joint was restricted in extension after the muscle transplantation; the active range of the joint was -30° in extension and 130° in flexion. The flexion force of the joint was augmented by the muscle transplantation and recovered to an M5 level. Recovery of touch sensation advanced to the base of the index and middle fingers and thumb. The sensation in the left palm was S2 on the MRCS. When the tips of the fingers and thumb were touched or scrubbed, he did not recognized it as a sensation of his left digits but as a weak tingling pain at his right middle fingertip. The sign of motor recovery of the left median nerve has not been observed yet.

As the final stage of the reconstruction, we plan to perform biceps brachii muscle transfer to the triceps muscle to stabilize the elbow joint, tenodesis at the wrist and arthrodesis of the thumb to obtain pinch function. The patient has not yet agreed to final operation, because he is completely satisfied with the improved muscle strength of the elbow flexion and is worried about possible loss of elbow flexion strength after the biceps brachii muscle transfer.

## Discussion

This patient with BPI involving the total brachial plexus had undergone intercostal nerve transfer [9] to the musculocutaneous nerve and a spinal accessory nerve transfer [10] to the suprascapular nerve in a previous hospital. We restored sensation of the palm and motion of the wrist to the patient by transplantation of a vascularized ulnar nerve between the CC7 and the median nerve and a free vascularized gracilis muscle innervated by a part of the transplanted vascularized ulnar nerve. Nineteen months after our first operation, the affected wrist was flexed dorsally by the transplanted muscle innervated by a part of the vascularized nerve connecting to the CC7 and touch sensation recovered up to the base of each fingers.

Several authors have reported excellent outcomes of the sensory and motor recovery of the median nerve using a vascularized nerve transfer connected to the CC7 [1,3,5,7]. Poor motor recovery is to be expected in muscles denervated for more than six months, and skin sensation does not recover if it has been denervated for a long time, because its muscle fibers or sensory organs become too atrophied [11,12]. In our patient, median nerve reconstruction was performed 16 months after the original injury. Excellent recovery of the sensory and motor function of the median nerve could not be expected in this patient, even with CC7 transfer to the median nerve. Thus, it was controversial whether this patient had a valid indication for a CC7 transfer to reconstruct the median nerve. Moreover, for this patient no motor source was available without the vascularized nerve connecting CC7. The intercostal nerves and the accessory nerve had already been used to restore elbow flexion and shoulder mobility. The patient had left phrenic nerve palsy. The patient had no chance of motor recovery without using a free functioning muscle transfer, as no muscles in the affected arm would function even after the neurotization because of the prolonged loss of innervation. Therefore, we decided to perform a CC7 transfer to the median nerve, expecting it to serve as the motor source of a free functioning muscle transfer as well as for recovery of the sensation of the hand. Because a movable hand lacking sensation is no more harmful than a totally paralyzed hand, we reached a consensus with the patient before surgery that the free gracilis muscle transfer would not be done if the sensory recovery of the median nerve was poor. Actually, the patient showed more rapid and better sensory recovery in the median nerve than we had expected before the CC7 operation. Four months after the operation, the patient recognized touch sensation up to the midpalm level of the hand. The sensory recovery of this patient was extremely faster than that reported by previous authors [1,3,5,7]. Although there may be different opinions [7], one possible explanation of the rapid nerve recovery through the CC7 operation in this patient is that the nerve recovery in the transplanted ulnar nerve might have been accelerated by sufficient vascular supply supercharged by the accompanying ulnar artery connected to the transverse cervical artery in the right neck. Most previous authors performed CC7 operations without supercharging the ulnar vessels accompanying the transplanted ulnar nerve [1,3,5]. In addition, the patient was still 22 years old when he had the CC7 operation. His youth might have also contributed to his rapid sensory recovery of his affected median nerve.

Several surgeons have treated patients with upper BPI using a part of the ulnar nerve transferred to the biceps brachii branch of the musculocutaneous nerve and have demonstrated rapid reinnervation of the biceps brachii muscles without leaving any functional loss in the donor ulnar nerve [2,4,6,10]. Oberlin et al. reported that no functional loss should occur in the ulnar nerve after partial ulnar transfer, because the nerve fibers are still mixed and their locations are not distributed functionally at the midarm level [2]. In our patient, no regression of the sensation of the affected hand was observed after a part of the vascularized nerve was transferred to a branch innervating the free transplanted muscle. A part of the transplanted ulnar nerve was taken in the subclavicular region and joined to the obturator nerve of the transplanted gracilis muscle. The ulnar nerve was sectioned just above the insertion of the superior ulnar collateral vessels to the ulnar nerve, and the distal portion of the nerve was reflected proximally in the CC7 operation. Thus, the portion where a part of the transplanted ulnar nerve had been harvested was almost the same as where a part of the ulnar nerve had been taken in Oberlin's original procedure [2]. Because the nerve fibers had not been distributed functionally in the portion where a part of the transplanted ulnar nerve was taken, the palmar sensation in this patient after harvesting a part of the transplanted nerve may not have been compromised.

In conclusion, part of the transplanted vascularized ulnar nerve connected to the contralateral healthy cervical seventh nerve root can be used successfully as a motor source and may be available in the treatment of patients with BPI with scanty motor sources.

## Competing interests

The author(s) declare that they have no competing interests.

## Authors' contributions

RK was responsible to this patient for the whole treatment of the brachial plexus injury and performed the surgery as a main surgeon. RI and KN assisted RK in the surgery. TN supervised the surgery and gave several suggestions to complete this manuscript. All authors read and approved the final manuscript.
